# SBA-15- and SBA-16-Functionalized Silicas as New Carriers of Niacinamide

**DOI:** 10.3390/ijms242417567

**Published:** 2023-12-17

**Authors:** Agata Wawrzyńczak, Izabela Nowak, Agnieszka Feliczak-Guzik

**Affiliations:** Faculty of Chemistry, Adam Mickiewicz University in Poznań, Uniwersytetu Poznańskiego 8, 61-614 Poznań, Poland; agata.wawrzynczak@amu.edu.pl (A.W.); nowakiza@amu.edu.pl (I.N.)

**Keywords:** amino-functionalized mesoporous silicas, carriers, niacinamide, protection, controlled release, functional foods

## Abstract

Amorphous silica as a food additive (E 551) is used in food materials (e.g., sweeteners, dairy products) for its anti-caking properties. The physicochemical properties of SiO_2_ also make it suitable to serve as a carrier of active substances in functional foods, dietary supplements, and drugs. Deficiency of niacinamide (vitamin B3, niacin) leads to several pathologies in the nervous system and causes one of the nutritional diseases called pellagra. The present study focuses on the use of hybrid ordered mesoporous silicas (SBA-15/SBA-16) functionalized with amino groups introduced through grafting or co-condensation with (N-vinylbenzyl)aminoethylaminopropyltrimethoxysilane (Z-6032) as novel carriers of niacinamide. They combine the characteristics of a relatively stable and chemically inert amorphous silica matrix with well-defined structural/textural parameters and organic functional groups that give specific chemical properties. The highest degree of carrier loading with niacinamide (16 wt.%) was recorded for the unmodified SBA-15. On the other hand, the highest degree of niacinamide release characterizes the functionalized SBA-15 sample (60% after 24 h), indicating that the presence of amino groups affects the release profile of niacinamide from the structure of the mesoporous silica.

## 1. Introduction

Nowadays, the human body is exposed to many harmful external factors. The ever-increasing pace of life, as well as the associated rush, stress, lack of exercise, sedentary work style, stimulants, and, above all, an improper and poorly balanced diet, increase the need for vitamins and minerals. Therefore, food fortification is very often considered by consumers as a necessary measure for proper functioning and maintenance of health. In the general sense, functional foods are foods that offer both basic nutrition as well as additional health benefits manifested in reducing the risk of chronic diseases. Functional foods can be divided into two groups: conventional and modified. Conventional ones include fruits, vegetables, nuts, seeds, herbs, spices, etc., whereas other products, such as fortified juices, grains, dairy products, and milk alternatives, are recognized as modified functional foods. However, it is important to note that these functional foods in no way replace a rational, well-balanced diet tailored to the needs of the body. Moreover, to improve the effectiveness of fortified food products, appropriate delivery systems are required to protect active ingredients from severe gastrointestinal tract conditions [1,2].

One of the components most commonly supplementing functional foods are vitamins. They are organic compounds necessary in small amounts in the human body, where they perform a variety of often complex functions. None of the vitamins can be omitted, as each is extremely important to maintaining our health [3]. The following article focuses on determining the impact of using modern carriers of active substances— namely, amino-functionalized nanoporous silicas with SBA-15- and SBA-16-type structures—on the release profile of vitamin B3 (niacin, pyridine-3-carboxylic acid).

The term “niacin“ is commonly used for the two basic free forms of vitamin B3— namely, nicotinic acid and nicotinic acid amide. Nicotinic acid and nicotinic acid amide, although they have the same vitamin properties, differ fundamentally in their effects on blood vessels and lipid metabolism: nicotinic acid protects against cardiovascular disease by dilating blood vessels, including the heart coronary vessels, and lowering plasma cholesterol and lipid levels. These effects, however, are not exhibited by nicotinic acid amide. Niacin deficiency leads to several pathologies in the nervous system, such as dementia or depression, and other symptoms similar to the neurodegenerative diseases [4]. Furthermore, deficiency of niacin is one of the causes of a nutritional disease called pellagra [5]. In recent years, the consumption of dietary supplements, including those containing vitamin B3, has increased significantly [6,7]. In addition to this, the possibility of providing additional vitamins with food products deserves attention. This role is played by functional foods, in which the base ingredient can also act as a carrier for active substances, such as vitamins. Some interest in designing a different delivery system for the controlled release of vitamin B3 may also be observed in the literature (e.g., [8,9]).

The synthesis, characterization, and application of nanoporous materials are still under extensive study. These materials are characterized by the presence of pores with diameters in the range of 1 nm to 100 nm [10,11,12]. They are highly diverse in terms of the composition, structure, and spatial organization of pores. Well-organized pores with nanoscale dimensions constitute a special nanostructure and consequently give the materials unique characteristics. Nanostructured porous materials include ordered micro- and mesoporous materials, pore-controlled glasses, as well as porous gels, polymers, and carbons [13,14].

Since 2001, when Vallet-Regi and co-workers [15] described the use of ordered mesoporous material of the MCM-41 type as a carrier in active substance delivery systems, great opportunities have opened up, mainly for pharmaceutical science. That work sparked the interest of the researchers and initiated the use of new silica-based mesoporous materials as carriers for selected active substances [16,17,18,19,20,21]. This trend may also be observed in the context of food ingredients [22,23,24,25,26], because the nanostructured amorphous silica has been positively evaluated as a food additive that does not cause any relevant systemic or local toxicity after oral exposure [1,27]. Furthermore, mesoporous silica-based materials have the potential to be used in food fortification as supports in smart delivery systems as they possess significant loading capacity, controlled release capability, and prolonged stability during digestion conditions. These materials are also characterized by good biocompatibility, especially when used in the form of microparticles [28].

In 1998, a new family of highly ordered mesoporous silica materials was synthesized following the use of non-ionic PEO–PPO–PEO (polyethylene polyoxide–propylene polyoxide–polyethylene oxide) triblock copolymers as surfactants [29]. The compounds obtained in this way were designated by the common term SBA. All representatives of this group of materials are characterized by similar features, including, namely, the presence of large pores with thick walls, a large specific surface area, and high hydrothermal stability. A variety of SBA materials has been described in the literature, with the important representatives being the silicas designated SBA-15 and SBA-16 (e.g., [30,31,32]).

The SBA-15 material has two-dimensional, hexagonally arranged channels with p6mm symmetry. The pores in this type of material reach sizes between 5 nm and 30 nm, while the specific surface area may be as high as 1100 m^2^/g. A major advantage of this type of silica is that it is characterized by thick walls in the range of 3–7 nm, which greatly improve the thermal and hydrothermal stability of SBA-15 when compared to other mesoporous silicas. The synthesis of SBA-15-type mesoporous silicas is based on triblock copolymers, which provide a template for polymerizing silica. For this purpose, Pluronic P123 (PEO)_20_(PPO)_70_(PEO)_20_ is most commonly used, while TEOS is the silicon source. The entire synthesis is carried out under strongly acidic conditions. It is worth mentioning that Pluronic P123, the structure directing agent used for SBA-15 matrix synthesis, is considered biodegradable and non-toxic [30].

SBA-15 is a material characterized by both microporosity and mesoporosity, where the network of micropores is a connector between the main mesopores. The microporosity results from partial deposition of the polyoxyethylene groups of the surfactant in the mesopore wall. The total microporosity of this material can even reach up to 30% of the total volume of all pores [33]. The size of micropores has been found to depend on the synthesis conditions and can vary from 0.5 nm to 2 nm. Interestingly, in the case of SBA-15 material, the final structure strongly depends on the conditions of the synthesis. It has been proven that parameters, such as temperature, pH, type of structure-directing agents, presence of electrolytes or salts, etc., allow for engineering the pore size and tuning the properties and morphology of SBA-15 to a great extent [34,35]. An additional advantage of all SBA materials (including SBA-15) synthesized using triblock copolymers is that the non-ionic surfactants are much easier to remove from the matrix due to their weaker interactions with inorganic silica.

On the other hand, the SBA-16 matrix can be obtained under acidic conditions following the use of triblock copolymers with longer chains than those in the Pluronic P123 surfactant, i.e., Pluronic F127 (PEO)_106_(PPO)_70_(PEO)_106_ [36]. For the synthesis of SBA-16, TEOS is most often used as the silicon source, whereas Pluronic F108 (PEO)_133_(PPO)_50_(PEO)_133_ or a mixture of P123 and F127 can be used as structure-directing agents, in addition to the aforementioned Pluronic F127. Due to the type of surfactants used, the described silica matrix can have up to 50% of micropores in its structure. SBA-16 silica materials are characterized by a narrow pore size distribution, mesopore sizes around 6 nm, a large specific surface area (reaching up to 1000 m^2^/g), large total pore volumes (0.3–0.6 cm^3^/g), and thick pore walls (4–6 nm) providing high chemical, thermal, and hydrothermal stability. The final morphology of the SBA-16 material can also be controlled by altering the time, temperature, pH, Si/surfactant ratio, surfactant type, co-surfactant addition, etc. [36]. Despite many similarities with SBA-15, the geometry and ordering of the pores in SBA-16 is different—it shows a cubic structure with Im3m symmetry and is characterized by a regular pore ordering, where each cage connects to neighboring cages through eight small slits, resulting in a three-dimensional, multidirectional network [37,38]. Additionally, it exhibits higher stability than SBA-15 silica, which is the result of thicker pore walls. According to a number of studies, the 3D structure of the SBA-16 material provides more favorable mass transfer kinetics when compared to the 2D network of the SBA-15 material.

To functionalize ordered mesoporous silica materials with amino groups, suitable organosilanes containing the desired functional groups are usually applied. They can be introduced both by co-condensation—that is, already during the synthesis of the mesoporous material—and by grafting onto the surface structure of the synthesized silica [39,40,41]. Aminopropylsilanes, such as (3-aminopropyl)triethoxysilane (APTES), are most commonly used precursors for modifying the surface of silica serving as a carrier of active substances for controlled release (e.g., [42,43,44]). In anhydrous media, the reaction with silica surface groups occurs by exchanging the methoxy or ethoxy groups present in the organosilane molecule for the oxygen atom of the silanol group present on the silica surface, resulting in the loss of the corresponding alcohol. The selection of a suitable aminoorganosilane is essential to control the amount, distribution, and stability of functional groups incorporated into mesoporous silica [45].

Within the framework of this work, research was conducted on the possibility of using pristine and aminosilane-modified silica materials with various structural and textural parameters as carriers for the gradual release of niacinamide with a potential application in fortified foods where silica (E 551) serves as an anti-caking agent. To the best of our knowledge, studies on the effect of the structure of the silica matrix on the release profile of niacinamide have not yet been widely conducted, nor has the effect of release conditions (different pH values, the presence of a promoter) been determined for such systems.

## 2. Results and Discussion

### 2.1. XRD

**Figure 1:**
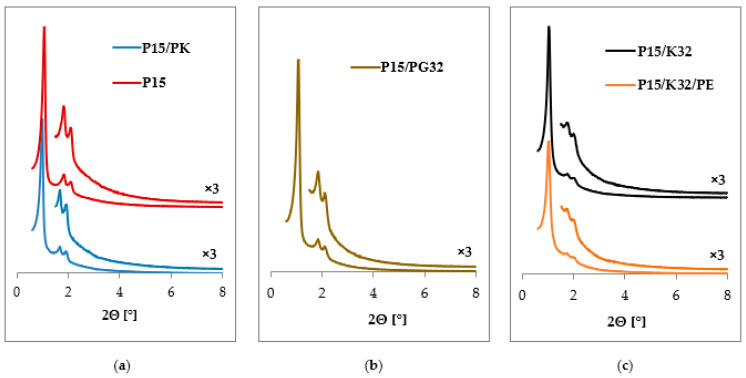
Diffractograms for SBA-15-based materials: (**a**) unmodified samples before and after calcination; (**b**) SBA-15 modified with Z-6032 aminoorganosilane through grafting; (**c**) SBA-15 modified with Z-6032 aminoorganosilane through co-condensation, before and after extraction.

XRD measurements in the range of low 2Θ angles were performed to obtain the information on the degree of structural ordering of amino-modified and unmodified mesoporous materials of the SBA-15 and SBA-16 types. The diffractograms presented in Figure 1a,c show the influence of the method applied for structure-directing agent (template) removal by means of calcination or extraction. In Figure 1b,c, the influence of the presence of aminoorganosilane on the SBA-15 porous structure has been shown.

The diffractograms shown in Figure 1 demonstrate the presence of three distinct and well-resolved reflections in the low-angle range of 2Θ. The reflections at 2Θ angles of 1.0°, 1.7°, and 1.9° are attributed to the (100), (110), and (200) indexes, respectively, and correspond to the hexagonal arrangement of pores in the spatial symmetry of p6mm [29]. As a result of the template removal from the silica matrix, the intensity of the reflections increases, which is the case for both methods (namely, calcination and extraction). When analyzing the diffractogram of the sample before and after calcination (Figure 1a), a shift of the maximum of the reflection peak towards higher values of 2Θ angles is observed, which may indicate a decrease in the interplanar distance due to the shrinkage of the mesoporous structure. The condensation of silanol groups inside the walls and on the surface of the material, which occurs at an elevated temperature, is responsible for this phenomenon [46].

Comparing the diffractograms of materials before and after surface modification, it can be seen that the material before the introduction of aminoorganosilane is characterized by the highest quality of mesopores ordering. Nevertheless, the samples modified with Z-6032 aminoorganosilane still have good structure ordering, which has been preserved regardless of the functionalization method (Figure 1b,c) [31,47]. In the case of the co-condensation method, aminoorganosilane is applied already during the siliceous matrix formation. Due to its presence, a gentler method for surfactant removal should be used. The diffractograms presented in Figure 1c show the effect of the extraction process on the structural ordering of the SBA-15 material modified with Z-6032. Its analysis allows us to conclude that the applied extraction procedure not only enables the removal of the surfactant from the matrix, but also preserves the ordered mesoporous structure.

**Figure 2:**
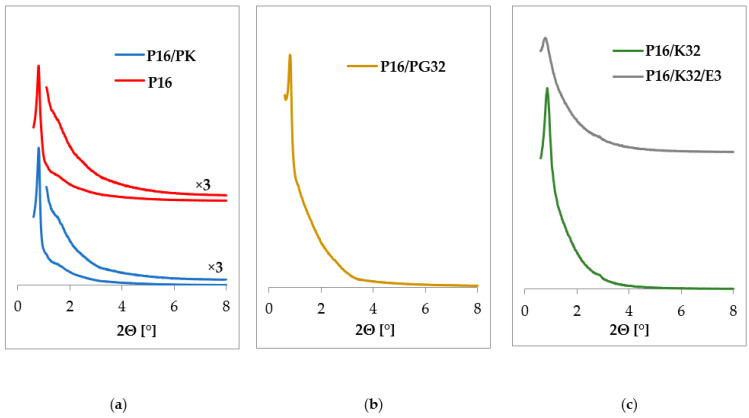
Diffractograms for SBA-16-based materials: (**a**) unmodified samples before and after calcination; (**b**) SB6-15 modified with Z-6032 aminoorganosilane through grafting; (**c**) SBA-16 modified with Z-6032 aminoorganosilane through co-condensation, before and after extraction.

Figure 2 presents diffractograms showing the influence of the template removal method (Figure 2a) and modification with aminoorganosilane (Figure 2b) on the structural ordering for the SBA-16-based materials. A typical diffractogram for an SBA-16 material with pores arranged in a regular structure with Im3m symmetry is characterized by the presence of reflections with (110), (200), and (211) indexes [29]. The diffractograms in Figure 2 reveal the presence of only the first reflection in the low-angle range at 2Θ of about 0.8°, corresponding to the (110). The absence of (200) and (211) reflections may indicate a slightly weaker ordering of the structure in the synthesized materials [31,47].

Removal of the template in the case of SBA-16 does not increase the intensity of the reflections as it did for the SBA-15 material. Diffractograms for samples before and after template removal look similar for both extraction and calcination procedures (Figure 2).

Modification of SBA-16 samples with aminoorganosilane Z-6032 leads to XRD patterns with more intense reflections for samples functionalized through grafting (Figure 2b) than for samples obtained through co-condensation (Figure 2c). The maximum of the reflection did not shift in any of the discussed samples. This may indicate that the chosen synthesis parameters allowed us to obtain amino-functionalized SBA-16 materials with well-preserved structure ordering [31,47].

### 2.2. FT-IR

FT-IR spectra were recorded for the synthesized samples (Figures S4–S9) and Z-6032 aminoorganosilane (Figure S2), as well as for surfactants Pluronic P123 and F127 (Figure S3). The bands present in the spectra of pristine and modified mesoporous materials of the SBA-15- and SBA-16-type were compared with those present in the spectra of aminoorganosilane and surfactants. Both structure-directing agents have characteristic bands in the same range of the wave number (from 3000 to 2750 cm^−1^ and from 1470 to 1340 cm^−1^).

The effect of calcination conditions on the template removal from the SBA-15 and SBA-16 materials can be observed on the respective spectra (Figures S4 and S7). At wavenumbers in the range of 3600–3100 cm^−1^, a broad band originating from silanol groups (Si-OH) and physically adsorbed water may be observed. It decreases after calcination, indicating that dehydration is taking place during this process. Additionally, in the spectra of the materials before calcination, a band is present at about 2956 cm^−1^, which may be attributed to stretching vibrations originating from the -CH_2_ and -CH_3_ groups of the structure-directing agents. Bands at a wavenumber of 1462 cm^−1^, corresponding to asymmetric vibrations of -CH_3_ from the template, and two weak bands at 1380 cm^−1^ and 1350 cm^−1^, attributed to symmetric and stretching vibrations stemming from -CH_3_ groups, respectively, are also present. All of the aforementioned signals disappear after the calcination process, indicating complete removal of the surfactant from the siliceous structure. The spectra also show intense bands at wavenumbers ranging from 1200 to 1000 cm^−1^, which may be attributed to two overlapping effects from stretching and deformation vibrations of the siloxane chains. At 805 and 955 cm^−1^, two bands characteristic of the stretching vibrations of non-bridging oxygen atoms (Si-O-) in Si-O-H bonds and the symmetric stretching vibrations in Si-O-Si can be also observed [48].

Figures S5, S6, S8 and S9 show FT-IR spectra for silicas after surface functionalization with Z-6032 aminoorganosilane. They also include materials before and after the extraction procedure. The overlapping of the bands corresponding to -CH_2_ and -CH_3_ stretching vibrations from the template and introduced aminoorganosilane may occur (e.g., in the spectra of samples P15/K32/E3, Figure S6 and P16/K32/E3, Figure S9). On both spectra— namely, for samples before removal of the template and after extraction—weak bands are present at 1633 cm^−1^, 1462 cm^−1^, 1380 cm^−1^, and 1350 cm^−1^. These bands may be from both the introduced amine and the residues of unextracted surfactant. However, the band at the wavenumber of 1462 cm^−1^ can be attributed to the deformation vibrations originating from secondary amines (Z-6032). On the other hand, the weak band at 1380 cm^−1^ can be attributed to C-H bending vibrations coming from the vinyl group present in the aminoorganosilane Z-6032. Thus, the results of FT-IR analysis confirmed the introduction of the amino groups into the structure of the mesoporous materials of SBA-15 and SBA-16 types. In addition, at 805 cm^−1^ and 955 cm^−1^, two bands attributed to characteristic stretching vibrations of non-bridging oxygen atoms (Si-O-) in Si-O-H bonds and symmetric stretching vibrations (Si-O-Si) can be observed. At the same time, bands originating from the aromatic ring substituted in the para position, present in the aminoorganosilane Z-6032, should appear in this region (810–840 cm^−1^) [47,48].

### 2.3. Low-Temperature Nitrogen Sorption Measurements

Low-temperature nitrogen adsorption/desorption measurements for textural characterization of the samples were carried out to determine the specific surface area (through the BET method), as well as the total volume and average size of pores. Table 1 summarizes the textural data for the unmodified and Z-6032-modified SBA-15 and SBA-16 materials.

Based on the obtained results, it can be seen that non-functionalized SBA-15 and SBA-16 materials are characterized by the largest specific surface area of more than 700 m^2^/g and a total pore volume of more than 0.60 cm^3^/g. The pore sizes calculated from the adsorption branch for materials modified with aminoorganosilane Z-6032 are comparable to those for pristine silicas. However, significant changes may be observed when non-functionalized materials are compared with their functionalized counterparts. This indicates that the introduction of Z-6032 aminoorganosilane can block pores of smaller sizes and make them inaccessible for nitrogen molecules, as well as for other reactants [49]. This is particularly visible for samples obtained via the co-condensation method.

**Figure 3:**
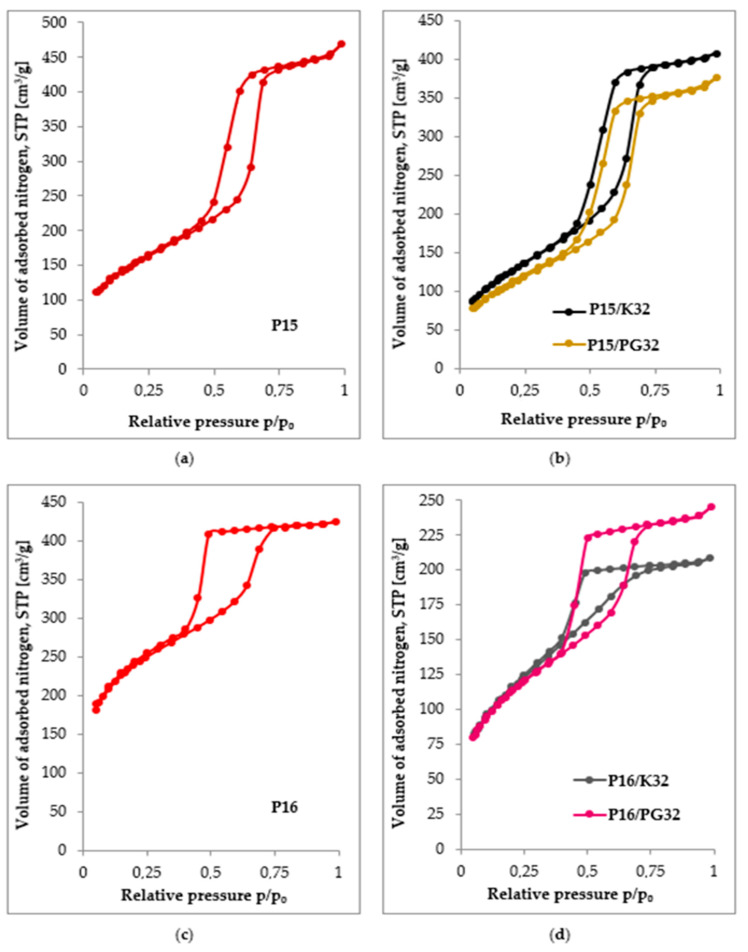
Nitrogen adsorption/desorption isotherms for: (**a**) unmodified SBA-15 material; (**b**) SBA-15 materials modified with Z-6032 through grafting and co-condensation; (**c**) unmodified SBA-16 material; (**d**) SBA-16 materials modified with Z-6032 through grafting and co-condensation.

The low-temperature N_2_ adsorption/desorption isotherms prove the mesoporous nature of the synthesized materials. Analysis of the results allows us to conclude that, according to the IUPAC classification, type IV(a) isotherms with H1 hysteresis loop are observed for materials based on SBA-15 silica (Figure 3a,b), indicating the presence of cylindrical pores [50].

On the other hand, type IV(a) isotherms with H2 hysteresis loop were obtained for SBA-16 materials (Figure 3c,d). This may indicate the presence of mesopores with narrow inlets and wider interiors (so-called “ink bottle shape” pores) [50]. The co-condensation method for silica functionalization results in a slightly distorted sorption isotherm, but a shape that can be described as consistent with type IV(a) is still preserved; thus, it can be concluded that the material obtained through this method is a mesoporous material with a pretty well-shaped structure (Table 1). Also, the surface modification achieved through the grafting method makes it possible to obtain an SBA-16 sample with a well-developed mesoporosity, as confirmed by both the appearance of adsorption/desorption isotherms (Figure 3d) and the textural data in Table 1.

### 2.4. TEM Analysis

Investigations performed using transmission electron microscopy allowed us to approximate the structures of the obtained ordered mesoporous materials of the SBA-15 and SBA-16 types (Figure S10). The photographs show very good ordering of the structure in both materials studied—namely, SBA-15 with a hexagonal structure of p6mm symmetry and SBA-16 with a regular structure of Im3m symmetry [31,47]. In both types of samples, the pore sizes are definitely below 10 nm, which is in full agreement with the data presented in Table 1. Furthermore, the high degree of structure ordering, which is typical of the SBA-15 and SBA-16 materials, was also confirmed by the results of XRD analysis (Figure 1 and Figure 2).

In the images of the amino-functionalized SBA-15 and SBA-16 (Figure S10C,D), an arrangement of pores characteristic of these types of materials was also observed. Thus, the application of aminoorganosilane to the surface of the silicas did not cause as major a disruption in their ordered structure as might have been expected, given, among other results, the results of XRD measurements.

### 2.5. Elemental Analysis

The elemental analysis allowed for the estimation of the amount of amino groups introduced during silica functionalization and the quantity of niacinamide deposited during the impregnation process. The elemental composition of the materials obtained is shown in Table 2.

From the data presented above, it can be seen that the highest average percent of nitrogen content for SBA-15 and SBA-16 materials occurs for samples modified with Z-6032 aminoorganosilane introduced through grafting—namely, 0.66 wt.% and 0.67 wt.% for samples P15/PG32 and P16/PG32, respectively. For both types of SBA materials, the least effective method of introducing amino groups turned out to be the co-condensation method, as nitrogen content reached only 0.26 wt.% and 0.14 wt.% for samples P15/K32 and P16/K32, respectively. This agrees well with previously reported data, where secondary amines were shown to be better modifying agents for grafting than their primary counterparts [45,51]. A comparison between SBA-15- and SBA-16-based materials shows that the grafting method allows for the introduction of a similar number of amino groups. However, this is not the case for the co-condensation procedure—here, the loading with amino groups is significantly lower in the P16/K32 material than in the P15/K32 sample. It can be concluded that the amount of nitrogen that is introduced into the mesoporous structure depends not only on the choice of modification method, but also on the structure of the material subjected to modification.

In addition, by observing an increase in the nitrogen content for materials impregnated with niacinamide solution, the deposition of this substance in the mesoporous structure of silica supports can be confirmed. The most pronounced increase in nitrogen content can be observed for unmodified silica samples, i.e., P15 + NA and P16 + NA. An increase in nitrogen content can be also noticed for aminoorganosilane-functionalized materials, and thus it may be concluded that niacinamide was also deposited in the mesopores of these materials. Moreover, a correlation could be observed when the increase in nitrogen content resulting from the elemental analysis is compared with the percent of loading values obtained from the HPLC measurements (Table 2).

### 2.6. Thermogravimetric Analysis

The results of the thermogravimetric analysis of pure and modified mesoporous carriers loaded with niacinamide are shown in Figure S11. The maximum weight loss observed around 250 °C corresponds to the thermal decomposition of niacinamide. This peak may be observed for all SBA-15-based samples; nevertheless, its intensity is the highest for the P15 + NA sample, which turned out to be the most efficiently loaded with niacinamide (Table 2). On the other hand, at 370 °C, decomposition of the introduced amine (Z-6032) is also observed [42]. In the case of samples modified with Z-6032 and containing niacinamide, both the main peak from the decomposition of the amine and a small shoulder can be observed at temperature values close to 370 °C and 250 °C, respectively. Such a profile indicates the simultaneous presence of amine and adsorbed niacinamide. In addition, when comparing the TG profiles of P15/PG32 + NA and P15/K32 + NA samples, the shoulder at 250 °C is more visible for the first sample, suggesting a greater amount of adsorbed niacinamide. This was also confirmed by the elemental analysis results (Table 2). Based on the data obtained, the presence of amine as a modifying agent of the silica surface and the successful loading of the samples with niacinamide were confirmed.

### 2.7. Niacinamide Loading and Release Profiles

The use of nanoporous materials—specifically, ordered mesoporous silica—as carriers can positively affect the rate of delivery of the active ingredient, thus enabling its gradual release [16,17,52,53]. Therefore, silica materials with different pore structures—namely, SBA-15 and SBA-16, both pristine and amine-modified—could serve as carriers for controlled delivery of niacinamide.

The surface chemistry of the silica-based materials strongly depends on the silanol groups. The presence of amino groups changes the charge behavior of the silicas’ surface. This is the pH-dependent phenomenon, and the amino groups should be almost fully protonated at pH values below 9. Therefore, the effective charge of the surface in the aminoorganosilane-functionalized silica is influenced by the pH and by the relative concentration of ionizable groups on the surface, including amino-functionalized and residual, non-functionalized silanol groups [54]. As a result, in polar solvents (such as water used in this study), the ionic interaction between the drug molecule and the amino-functionalized silica carrier is also possible, and this may play a vital role in the drug adsorption and release. Moreover, in the case of the in vitro drug release, the polarity of the surface becomes an important parameter that significantly affects the diffusion of water into the porous structure of the carrier. The presence of aminoorganosilane molecules certainly lowers the polarity of the silica’s surface. Thus, a longer contact time with the drug solution is required in order to ensure an adequate degree of drug loading. On the other hand, lower polarity allows for a gradual release of the adsorbed drug to the polar (water) environment. Therefore, by increasing the hydrophobicity and reducing the wetting effect of the carrier, the unfavorable phenomenon of initial burst release of the drug can be reduced.

The values of niacinamide loading indicate that the highest amount of NA was adsorbed in the case of the unmodified silica materials P15 and P16, where the loading was as high as 16.3 wt.% and 9.3 wt.%, respectively (Table 2). On the contrary, the amine-modified samples had a significantly lower loading percentage when compared to their unmodified counterparts. This may indicate a preference of NA for interacting directly with the silanol groups present on the surface of the silica carrier rather than amine-mediated binding. This was particularly evident in the case of the sample P15/K32 + NA, where amine was introduced through co-precipitation and the percentage of NA loading reached only 3.0 wt.%. This is quite surprising, especially in light of previous studies on SBA-15 modified with sulfonic groups, where the introduction of functional groups improved the sorption capacity of the silica material [50]. It is likely that the lower amount of niacinamide introduced during our study is due to the fact that the amine introduced through co-precipitation caused partial blockage of the smaller mesopores and micropores connecting the mesoporous channels of the carrier, thus lowering the possibility of transporting niacinamide to the deeper parts of the carrier. This can be evidenced by the increase in the average pore size from 5.0 nm to 5.4 nm (Table 1), which confirms the blocking of smaller pores and making them inaccessible for the transport process and loading of the active substance. The blocking effect of Z-6032 aminoorganosilane on the possibility of niacinamide deposition in the silica structure is more pronounced for P15-based than for P16-based materials, confirming the beneficial effect of the three-dimensional pore arrangement in the silica matrix. In the case of the P16 carrier, even if the smaller pores are partially blocked by aminoorganosilane molecules, the three-dimensional channel network is well-developed, and transportation of niacinamide to the deeper parts of the carrier is still possible.

NA release studies were conducted using different acceptor fluids that simulated the pH of different parts of the gastrointestinal tract, i.e., pH 3.5 corresponds to the stomach environment and pH 7.4 is similar to the pH of the small intestine. In addition, modification of the acceptor fluids through the addition of EtOH was used to test the effect of the presence of the release promoter on the rate of NA release. On the basis of UV-Vis analysis performed for the blanks, it can be concluded that Z-6032 aminoorganosilane as a modifying agent was permanently bound to the siliceous carrier and did not affect the determination of the release profiles of niacinamide.

**Figure 4:**
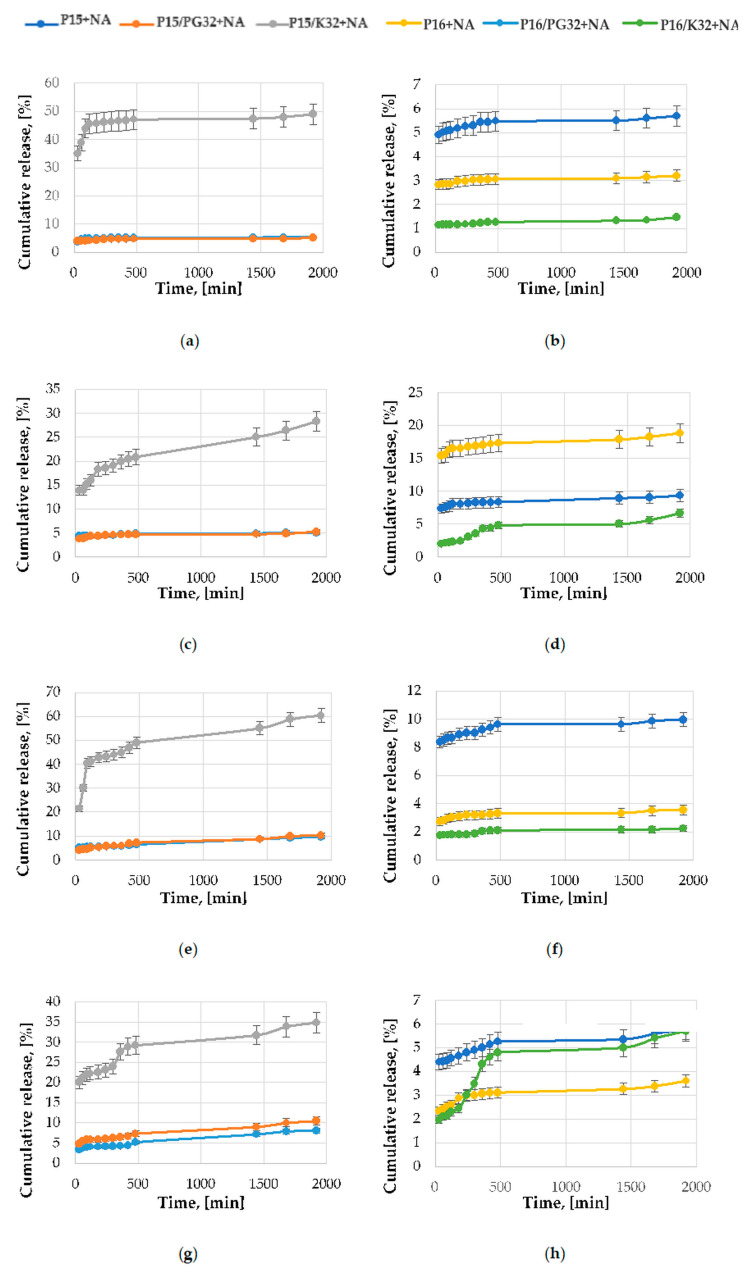
Release profiles in different types of acceptor media for silica samples loaded with niacinamide (**a**,**b**) acetate buffer (3.5); (**c**,**d**) acetate buffer with ethanol (3.5 + EtOH); (**e**,**f**) borate buffer (7.4); (**g**,**h**) borate buffer with ethanol (7.4 + EtOH).

Figure 4 shows cumulative release profiles obtained on the basis of HPLC determination of NA present in different acceptor media after 24 h of testing.

Different release profiles were observed for various types of carriers, which further varied depending on the pH of the acceptor fluid used. The fact that niacinamide in the sample P15/K32 + NA was deposited mainly in the outer regions of the pores resulted in the highest levels of release of the active substance for this material (Figure 4). On the contrary, for the sample P16/K32 + NA, the release profiles indicate stronger interactions of niacinamide with the carrier, as the amounts of active substance released are the lowest of all the samples tested. This most likely indicates deposition of the active substance in the deeper parts of the carrier’s mesoporous structure. On the other hand, samples prepared on the basis of the SBA-16 material functionalized with amine groups introduced through grafting (P16/PG32 + NA) show a higher release percentage of the loaded niacinamide to the acceptor fluid (Figure 4).

It is also noteworthy that in the case of the sample with the highest initial loading of niacinamide (P15 + NA), only a small part of the applied active substance is released from the carrier into the acceptor fluid, as evidenced by the low value of the R parameter, i.e., the ratio between maximum value of cumulative release (after 24 h) and loading of niacinamide (Table 2). Slightly better values of this parameter for the P15 + NA sample can be observed when acceptor medium with higher pH is used.

Ethanol is frequently used in various pharmaceutical and cosmetic formulations to increase the skin permeability of active ingredients. Although ethanol is known to distribute throughout the skin and promote the penetration of polar and non-polar molecules, the exact mechanism by which it increases skin permeability is not fully understood. The penetration-enhancing effect of ethanol is due to its excellent ability to form hydrogen bonds with the head group atoms of skin lipids [17,55].

The addition of EtOH to the acceptor fluid promotes the release of niacinamide from deeper parts of the carrier, which can be observed in particular for the P16/K32 + NA sample. In contrast, the presence of EtOH reduces the release of niacinamide adsorbed in the outer parts of the mesoporous structure of the P15/K32 + NA material (Figure S12). It can also be observed that for acceptor fluid at pH 3.5, the effect of the presence of EtOH on the release profile of niacinamide is more pronounced than for systems based on the acceptor fluid at pH 7.4.

It is worth noting that in the case of silica carrier modified with aminoorganosilane, the process of adsorption and release of the drug will be significantly affected by the pH of the environment. This parameter determines, among other things, the degree of protonation of surface silanol and amine groups and thus adsorbate–adsorbent interactions. Lower pH values favor deprotonation of silanol groups, which can weaken the hydrogen bonds and electrostatic interactions formed between the niacinamide molecule and the surface of the carrier. This may be one of the reasons for the increased release of niacinamide in acidic media. Similar observations were made by Santha Moorthy et al. in their study on the pH stimuli-responsive mesoporous organosilica hybrid carrier for anticancer (5-fluorouracil) and anti-inflammatory (ibuprofen) drugs, where a lower pH value caused a high degree of protonation of the surface pyridyl groups and 5-fluorouracil molecules, resulting in strong electrostatic repulsion between the drug and binding sites of the carrier. Therefore, an enhanced release rate of 5-fluorouracil could be observed at pH = 5.5 [56]. This phenomenon is very important for the pH-responsive release performance under acidic pH environments in the presence of a triggering environment (e.g., an alternating magnetic field, as in [20]).

Mathematical models are commonly used to simplify the complex release process and gain insight into the release mechanisms of a specific material system. Methods depending on different mathematical models (for example, zero-order, first-order, Higuchi, and Korsmeyer–Peppas models) are usually used to study the drug release mechanism [57]. The drug-release values obtained during our study were substituted into the appropriate formulas, and the results were subjected to linear function fitting to obtain the corresponding values of the R^2^ parameter (Table 3). The correlation coefficient values were used as the criteria in selecting the best model for studying the drug release kinetics. Considering the data obtained for samples with the highest degrees of niacinamide release, i.e., P15/K32 + NA and P16/PG32 + NA, it can be concluded that R^2^ values for the Higuchi model are greater than those for the zero-order and first-order model, indicating that the niacinamide release from the aforementioned silica samples is controlled by a diffusion process [58]. Moreover, the best fitting is obtained for the Korsmeyer–Peppas model, where the data obtained from in vitro drug release studies were plotted as ln cumulative percentage of drug release versus ln time. The slope (n) and intercept (k) of this linear plot are the diffusion exponent and the kinetic constant, respectively. The diffusion exponent (n), which is a measure of the primary mechanism of drug release, corresponds to a Fickian diffusion mechanism when n ≥ 0.45, whereas n < 0.89 indicates anomalous (non-Fickian) diffusion. According to our results, the niacinamide dissolution process from aminoorganosilane-functionalized SBA-15 and SBA-16 samples follows a Fickian diffusion mechanism.

In conclusion, the slower release rate of niacinamide was demonstrated by carriers prepared on P16 material, which is characterized by a three-dimensional arrangement of mesopores. A similar observation was made by García-Muñoz and co-workers, who investigated the possibility of using mesoporous carriers with different structures for the controlled release of methylprednisolone hemisuccinate [59]. In the three-dimensional structure, the adsorbed active ingredient has the opportunity to diffuse into deeper parts of the carrier, causing the subsequent release of the active ingredient to be slowed down. This can play a positive role, especially when it is necessary to obtain carriers for formulations with prolonged action. As a result, it appears that these materials may be potential carriers beneficial for niacinamide supplementation in functional food products.

## 3. Materials and Methods

### 3.1. Synthesis of Porous Materials of SBA-15 Type

The synthesis consisted of weighing 2 g of Pluronic 123 (PEO_20_PPO_70_PEO_20_ triblock copolymer, 98 %, BASF, West Port Arthur Road, Beaumont, TX, USA) in the polypropylene (HDPP) bottle and dissolving it in 60 cm^3^ of HCl (2 mol/dm^3^, POCh, Poznan, Poland) at room temperature. Then, 8.8 g of sodium chloride (NaCl, 99.9 %, POCh, Poznan, Poland) and 4.2 g of tetraethyl orthosilicate (TEOS, 98 %, Sigma-Aldrich, Poznan, Poland) were added during vigorous stirring on a magnetic stirrer. The molar ratio of TEOS:P123:HCl (2 M): NaCl was kept as follows: 1.0:0.017:6.0:5.9. Stirring was continued at room temperature for 24 h, and then the reaction mixture was transferred to an oven and heated for 24 h at 100 °C. The product was filtered and washed with 500 cm^3^ of distilled water, followed by washing with 200 cm^3^ of ethanol (EtOH, 99.6 %, POCh, Poznan, Poland) to remove unreacted surfactant and sodium chloride. The product was then dried at 60 °C for 20 h and calcined at 550 °C for 6 h in order to remove the surfactant.

#### 3.1.1. Functionalization of SBA-15 Surface through Grafting

During the grafting procedure, the amino groups were introduced into the surface of the siliceous material by mixing 0.17 g of SBA-15, 16.7 cm^3^ of toluene (99.5 %, POCh, Poznan, Poland), and 0.093 cm^3^ of (N-vinylbenzyl)aminoethylaminopropyltrimethoxysilane (Z-6032, 40 % in MeOH, Dow Corning, Dow Center, Midland, MI 48674, USA, Figure S1). The proportions of SBA-15 and Z-6032 were chosen so that the molar ratio of Si:N was 1:0.3. The reaction mixture was stirred and heated on a magnetic stirrer at 90 °C for 24 h under a nitrogen atmosphere. The resulting product was then filtered, washed with toluene to remove the excess of the amine, and dried in the vacuum dryer at 80 °C for 24 h in order to eliminate the residues of the solvent from the pores of the silica.

#### 3.1.2. Functionalization of SBA-15 Surface through the Co-Condensation Method

The beginning of the procedure was analogous to the synthesis of pure SBA-15 described in Section 3.1. The difference was that 1 h after the introduction of TEOS, 0.264 cm^3^ of Z-6032 aminoorganosilane was added. The molar ratio of the resulting reaction mixture was as follows: TEOS:P123:HCl (2 M):NaCl:Z6032 = 1.0:0.017:6.0:5.9:0.049. The amount of Z-6032 was chosen so that the molar ratio of Si:N was 1:0.3. The reaction mixture was stirred for 24 h at room temperature and then placed in an oven and heated for 24 h at 100 °C. The resulting product was filtered, washed with 500 cm^3^ of distilled water and 200 cm^3^ of ethanol to remove the excess of the reactants, and dried at 60 °C for 20 h. Next, the material was subjected to the extraction process to remove the template, as calcination was not possible due to the presence of aminoorganosilane. In total, 0.5 g of the functionalized silica was mixed with 50 cm^3^ of 0.1 M HCl solution in EtOH and placed in an ultrasonic bath at 65 °C for a period of 3 h. After extraction, the product was filtered, washed with EtOH, air-dried, and ground in a mortar into a homogeneous powder.

### 3.2. Synthesis of Porous Materials of SBA-16 Type

The synthesis consisted of dissolving 1.07 g of Pluronic F127 (PEO_106_PPO_70_PEO_106_ triblock copolymer, 98 %, BASF, West Port Arthur Road, Beaumont, TX, USA) in a solution prepared by mixing 2.27 cm^3^ of HCl (36 %, POCh, Poznan, Poland) and 51.44 cm^3^ of distilled water. The solution was stirred and heated at 50 °C on a magnetic stirrer. After 0.5 h, 3.96 cm^3^ of n-butanol (BuOH, 99 %, POCh, Poznan, Poland), and, 1 h later, 5.46 cm^3^ of TEOS were added to the solution. The molar ratio of the reactants was: TEOS:F127:HCl:H_2_O:BuOH = 1.0:0.003:0.101:119.1:1.8. The solution was stirred and heated at 50 °C for 24 h and then transferred to an oven and heated at 100 °C for another 24 h. The final product was filtered and washed with 500 cm^3^ of distilled water and 200 cm^3^ of ethanol. Then, it was dried at 60 °C for 20 h and calcined at 550 °C for 6 h.

#### 3.2.1. Functionalization of SBA-16 Surface through Grafting

The procedure was analogous to that described for the SBA-15 material in Section 3.1.1.

#### 3.2.2. Functionalization of SBA-16 Surface through the Co-Condensation Method

At the beginning, the procedure was analogous to that described for non-modified SBA-16 material (Section 3.2). The difference was that 1 h after adding TEOS, 0.44 cm^3^ of Z-6032 amine was introduced into the reaction mixture. The molar ratio of reagents was as follows: TEOS:F127:HCl:H_2_O:BuOH:Z-6032 = 1.0:0.004:0.110:130.0:2.0:0.053. The amount of Z-6032 was chosen so that the molar ratio of Si:N was 1:0.3. The solution was stirred and heated at 50 °C for 24 h and then transferred to an oven and heated at 100 °C for another 24 h. The final product was filtered and washed with 500 cm^3^ of distilled water and 200 cm^3^ of ethanol. Then, it was dried at 60 °C for 20 h and calcined at 550 °C for 6 h. Next, the material was subjected to the extraction procedure in order to remove the template. This proceeded analogously to the one described for SBA-15 sample functionalized through co-condensation (Section 3.1.2).

### 3.3. Physicochemical Characteristics of the Materials Obtained

Physicochemical, structural, and textural parameters of the ordered mesoporous silicas were thoroughly characterized using several techniques—namely, X-ray diffraction (XRD), infrared spectroscopy (FT-IR), transmission electron microscopy (TEM), low-temperature nitrogen adsorption–desorption measurements, elemental analysis, and thermogravimetric analysis.

#### 3.3.1. XRD Analysis

A Bruker AXS D8 Advance (Billerica, MA, USA) diffractometer with a Johansson monochromator was used for the low-angle XRD measurements. The CuKα radiation source allowed wavelengths of λ = 0.154 nm to be obtained. The low-angle measurements included 2Θ values from 0.6° to 8.0°. The sample scan time was 3 s per measurement step of 0.02° 2Θ.

#### 3.3.2. FT-IR

FT-IR studies were conducted on an Agilent Technologies Cary 630 FTIR (Santa Clara, CA, USA) instrument using the attenuated total reflectance (ATR) technique. The measurement range was 4000 650 cm^−1^ while maintaining a resolution of 16 cm^−1^.

#### 3.3.3. Transmission Electron Microscopy (TEM)

TEM analysis was performed using a JEOL 200 CX (Tokyo, Japan) microscope operating at the 80 kV electron beam.

#### 3.3.4. Low-Temperature N_2_ Sorption Measurements

Low-temperature nitrogen adsorption/desorption measurements were performed using the Nova 1200 e analyzer (Quantachrome Instruments, Boynton Beach, FL, USA). Before analysis, samples were degassed for 24 h at 90 °C for materials functionalized with aminoorganosilane or at 300 °C for the pristine silica materials. Then, the measurements of nitrogen adsorption/desorption isotherms were carried out at the boiling point of this gas (−196 °C). From the data obtained, the specific surface (BET), total pore volume, and average pore size were determined.

#### 3.3.5. Elemental Analysis

Analysis of the elemental composition of the synthesized materials was performed on a FLASH 2000 (Thermo Scientific, Waltham, MA, USA) apparatus. It uses the method of the complete dynamic combustion of samples in an electronically temperature-controlled reduction–oxidation furnace. Each sample was measured in triplicate, and the obtained results were averaged.

#### 3.3.6. Thermogravimetric Analysis

The thermogravimetric analysis (DTA/TGA) was performed using the Netzsch TG 209 Libra (Selb, Germany) apparatus in an inert gas atmosphere and at a heating rate of 5 °C/min, with temperatures ranging from 20 °C to 800 °C.

### 3.4. Active Substance Loading

First, 200 mg of the carrier (amine-functionalized or pristine mesoporous silica) was poured with 5 cm^3^ of water, and then 120 mg of niacinamide (NA, 99.0 %, Fluka, Warsaw, Fluka) was added. The suspension was stirred with a magnetic stirrer for 24 h at ambient temperature (25 °C). Finally, the product was filtered and air-dried. Once the impregnation was completed, the loading percentage of the active substance was calculated through HPLC analysis. For this purpose, the initial solution and the solution after impregnation and filtering off the carrier were analyzed. From this difference, the % of loading of silica with niacinamide was calculated using the calibration curves. This research was conducted on the basis of our previous experiments, the results of which are partially described in the following papers [60,61].

### 3.5. Release Profiles of Niacinamide

First, 20 mg of the carrier loaded with niacinamide was weighed into the glass vial, and 15 cm^3^ of acceptor fluid (acetate buffer with pH 3.5 or borate buffer with pH 7.4) was added. In the case of the modified acceptor fluid, the mixture of the respective buffer and release promoter (EtOH) was used in a volumetric ratio of 4:1. The process was carried out at room temperature for 24 h with 100 µl samples taken every 30 min during the first 2 h and then taken every 60 min during the next 6 h. Each sample for the release profile measurements was duplicated in order to check the reproducibility, and the obtained results were averaged. During niacinamide release studies, blanks were also tested to eliminate the influence of possible leaching of amine from the silica carrier. For this purpose, analogous measurements of the release profiles were performed for amine-modified silicas without niacinamide loading.

The amount of the released active substance was determined through HPLC analysis using a 920-LC apparatus (Varian, Inc., Palo Alto, CA, USA) with a Microsorb MV 100-5 C8 250 × 4.6 mm column. The composition of the mobile phase was MeOH/1 % CH_3_COOH (70/30), and the flow rate was 1 cm^3^/min. The UV-Vis detector was operating at the wavelength corresponding to the maximum absorbance of the niacinamide (namely, 262 nm).

### 3.6. Samples Labelling

The materials discussed in this paper have been labelled as follows: P15 or P16 stand for pristine SBA-15 or SBA-16 silicas (Table 4). Samples modified with Z-6032 aminoorganosilane through grafting or the co-condensation method are labelled P15/PG32 (P16/PG32) or P15/K32 (P16/K32), respectively. Additional symbols, such as PE or PK, stand for the “prior-extraction” or “prior-calcination”, respectively, and relate to the method applied in order to remove the structure-directing agent. Samples loaded with niacinamide are marked with a +NA symbol.

## 4. Conclusions

The presented research aimed to determine the possibility of using modified SBA-15 and SBA-16 materials as carriers for niacinamide, e.g., in functional food products. The possibility of using them in the release process into acceptor fluids of different pH values and supported by the release promoters was also evaluated

In the course of this study, it was shown that the proposed procedures for the synthesis of SBA-15- and SBA-16-type mesoporous silicas functionalized with a secondary amine allowed us to obtain materials with structural and textural parameters characteristic of these types of carriers. The three-dimensional structure of mesopores in the samples based on amino-functionalized SBA-16 promotes the deposition of the active substance in the pores with a deeper location, but it hinders its release. In turn, it was proved that the addition of EtOH to the acceptor fluid promotes the release of niacinamide from deeper parts of the carrier. The addition of the promoter was also shown to have a greater effect on the release profile in the acceptor fluid with lower pH values.

Principally, a satisfying release rate of niacinamide was achieved when the surface of SBA-15 had been functionalized with amino groups introduced through the co-condensation method. The results of this study provide promising perspectives for the application of mesoporous materials based on SBA-15 and SBA-16 silicas as carriers of niacinamide in functional food products.

## Figures and Tables

**Table 1:** Textural data for pristine and aminoorganosilane-modified SBA-15 and SBA-16 materials.

Sample	Surface Area (BET) (m^2^/g)	Average Pore Size (BJH, ads. Branch) (nm)	Average Pore Size (BJH, des. Branch) (nm)	Total Pore Volume (cm^3^/g)	Average Pore Size (nm)
P15	717	6.4	4.9	0.89	5.0
P15/PG32	408	6.3	4.3	0.58	5.7
P15/K32	469	6.3	4.3	0.63	5.4
P16	806	6.3	3.8	0.63	3.1
P16/PG32	416	6.4	3.8	0.38	3.7
P16/K32	421	6.4	3.4	0.33	3.1

**Table 2:** Results of the analysis of elemental composition of the synthesized samples.

Sample	N, %	C, %	H, %	NA Loading, %	R			
					3.5	3.5 + EtOH	7.4	7.4 + EtOH
Niacinamide (NA)	22.46	58.43	5.00	-	-	-	-	-
P15	-	1.51	1.01	-	-	-	-	-
P15 + NA	2.50	8.01	1.57	16.30	0.33	0.31	0.59	0.49
P15/PG32	0.66	10.50	2.07	-	-	-	-	-
P15/PG32 + NA	1.62	13.00	2.28	6.30	0.82	0.83	1.65	1.66
P15/K32	0.26	11.33	2.45	-	-	-	-	-
P15/K32 + NA	0.72	13.20	2.61	3.00	16.33	9.44	20.13	11.62
P16	-	1.42	1.60	-	-	-	-	-
P16 + NA	1.42	4.93	1.09	9.30	0.34	2.03	0.38	0.39
P16/PG32	0.67	10.17	1.91	-	-	-	-	-
P16/PG32 + NA	1.82	11.56	1.95	7.50	0.76	1.25	1.33	0.77
P16/K32	0.14	10.12	2.41	-	-	-	-	-
P16/K32 + NA	1.07	12.63	2.63	6.30	0.23	1.06	0.36	0.91

NA loading—percentage of niacinamide on the silica surface, calculated on the basis of HPLC measurements. R—ratio between maximum value of cumulative release (after 24 h) and loading of niacinamide.

**Table 3:** Correlation coefficients (R^2^) for linear fit in kinetic models of niacinamide release from aminoorganosilane-functionalized SBA-15 and SBA-16 mesoporous silicas in different dissolution media.

Sample	Kinetic Model	Dissolution Medium			
		3.5	3.5 + EOH	7.4	7.4 + EtOH
P15 + NA	zero-order	0.2935	0.8474	0.9953	0.9827
	first-order	0.2951	0.8479	0.9956	0.9833
	Higuchi	0.4287	0.9260	0.9621	0.9573
	Korsmeyer–Peppas	0.6246	0.9006	0.8286	0.8552
P15/PG32 + NA	zero-order	0.5836	0.6151	0.9422	0.9747
	first-order	0.5845	0.6167	0.9455	0.9762
	Higuchi	0.7311	0.7535	0.9837	0.9781
	Korsmeyer–Peppas	0.8656	0.8893	0.9596	0.9128
P15/K32 + NA	zero-order	0.3301	0.8974	0.6900	0.8463
	first-order	0.3539	0.9122	0.7918	0.8615
	Higuchi	0.4826	0.9733	0.8177	0.9277
	Korsmeyer–Peppas	0.6984	0.9787	0.8604	0.9219
P16 + NA	zero-order	0.7230	0.8265	0.7079	0.7140
	first-order	0.7234	0.8308	0.7089	0.7155
	Higuchi	0.8517	0.9201	0.8470	0.8491
	Korsmeyer–Peppas	0.9301	0.9612	0.9592	0.9468
P16/PG32 + NA	zero-order	0.6923	0.8677	0.7489	0.8252
	first-order	0.6931	0.8699	0.7505	0.8264
	Higuchi	0.8376	0.9467	0.8786	0.9219
	Korsmeyer–Peppas	0.9488	0.9645	0.9498	0.9379
P16/K32 + NA	zero-order	0.8973	0.7983	0.7320	0.7170
	first-order	0.8974	0.8016	0.7324	0.7202
	Higuchi	0.9172	0.8882	0.8369	0.8426
	Korsmeyer–Peppas	0.8459	0.9108	0.8569	0.8942

**Table 4:** Labelling of materials used in this work.

Material	Labelling
P15	SBA-15
P16	SBA-16
P15/PG32	SBA-15 modified with Z-6032 aminoorganosilane through grafting
P16/PG32	SBA-16 modified with Z-6032 aminoorganosilane through grafting
P15/K32	SBA-15 modified with Z-6032 aminoorganosilane through co-condensation
P16/K32	SBA-16 modified with Z-6032 aminoorganosilane through co-condensation
PE	prior-extraction
PK	prior-calcination
P15/PG32 + NA	SBA-15 modified with Z-6032 aminoorganosilane through grafting loaded with niacinamide
P16/PG32 + NA	SBA-16 modified with Z-6032 aminoorganosilane through grafting loaded with niacinamide
P15/K32 + NA	SBA-15 modified with Z-6032 aminoorganosilane through co-condensation loaded with niacinamide
P16/K32 + NA	SBA-16 modified with Z-6032 aminoorganosilane through co-condensation loaded with niacinamide

## Data Availability

The data presented in this study are available upon request.

## Supplementary Materials

The supporting information can be downloaded at: https://www.mdpi.com/article/10.3390/ijms242417567/s1.
