# Supplementary material for: SBA-15- and SBA-16-Functionalized Silicas as New Carriers of Niacinamide
Source: Int J Mol Sci. 2023 Dec 17;24(24):17567. doi: 10.3390/ijms242417567 (PMC10743396; doi:10.3390/ijms242417567)
Supplement: Supplementary file 1 [file ijms-24-17567-s001.zip › ijms-2749623-supplementary.pdf]

SUPPORTING MATERIALS

SBA-15 and SBA-16 functionalized silicas as new carriers of niacinamide

Agata Wawrzyńczak[1], Izabela Nowak[1], Agnieszka Feliczak-Guzik[1*]

[1]Adam Mickiewicz University, Faculty of Chemistry, Uniwersytetu Poznańskiego 8, 61-614 Poznań, Poland

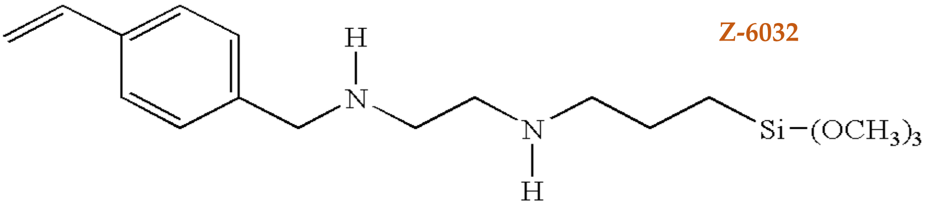

Figure S1. The chemical structure of (N-Vinylbenzyl)aminoethylaminopropyltrimethoxysilane (Z-6032).

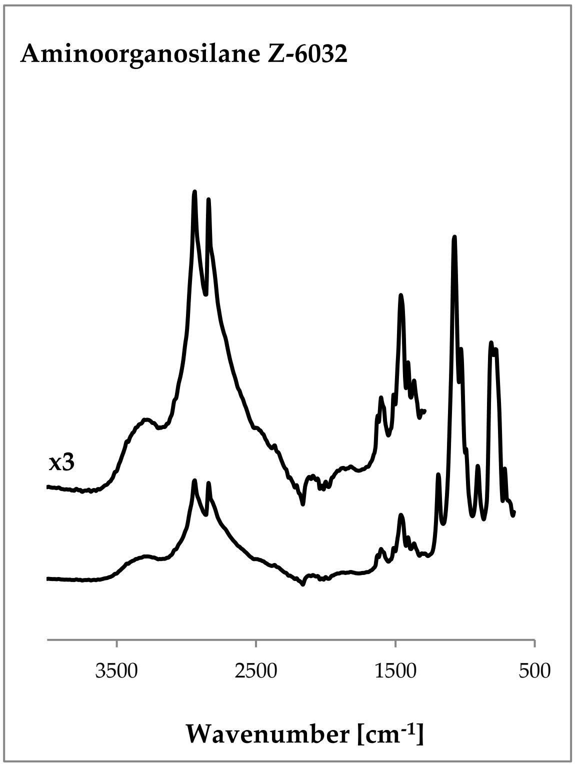

Figure S2. FT-IR spectra of Z-6032 aminoorganosilane.

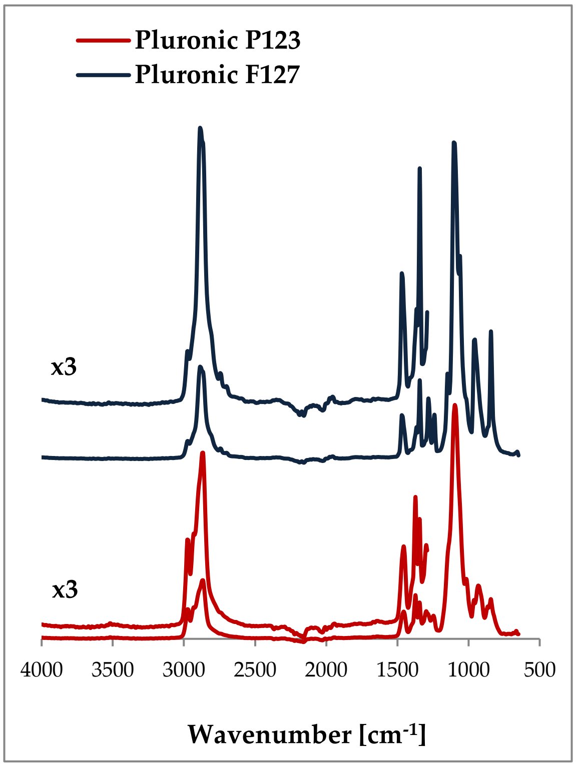

Figure S3. FT-IR spectra of Pluronic P123 and Pluronic F127.

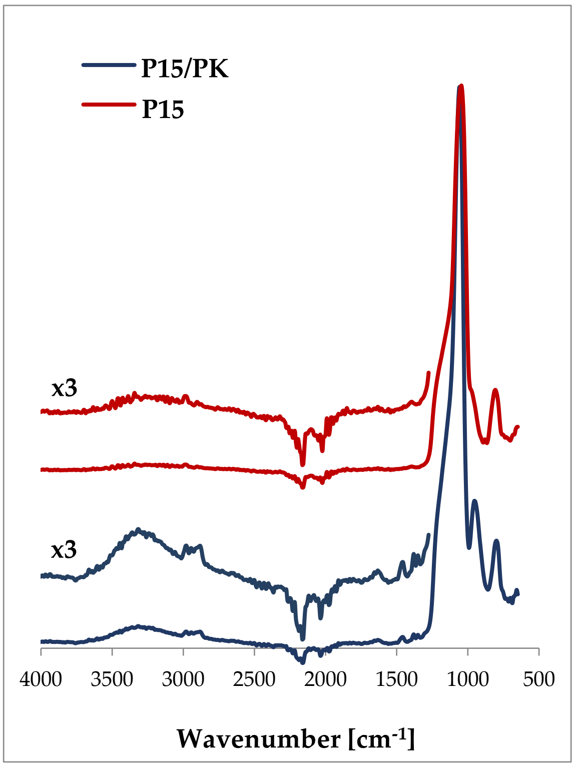

Figure S4. FT-IR spectra of as-synthesized and calcined SBA-15 materials.

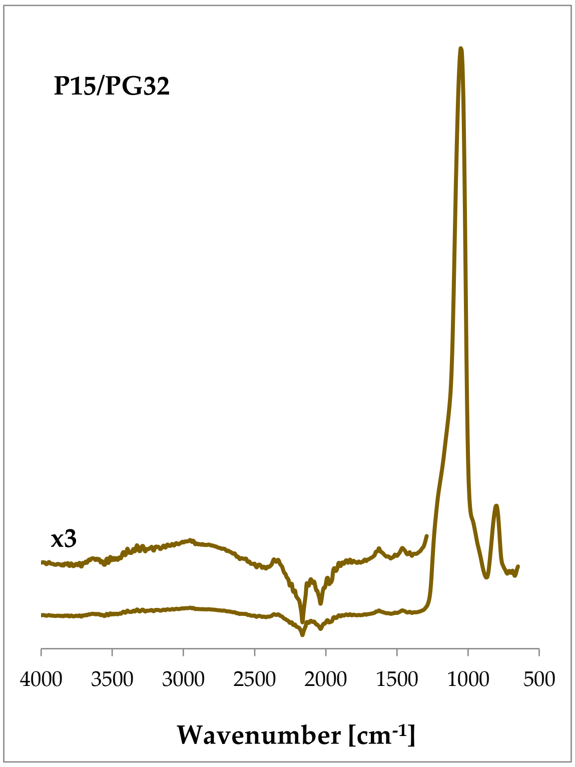

Figure S5. FT-IR spectra of SBA-15 material functionalized by grafting of Z-6032 aminoorganosilane.

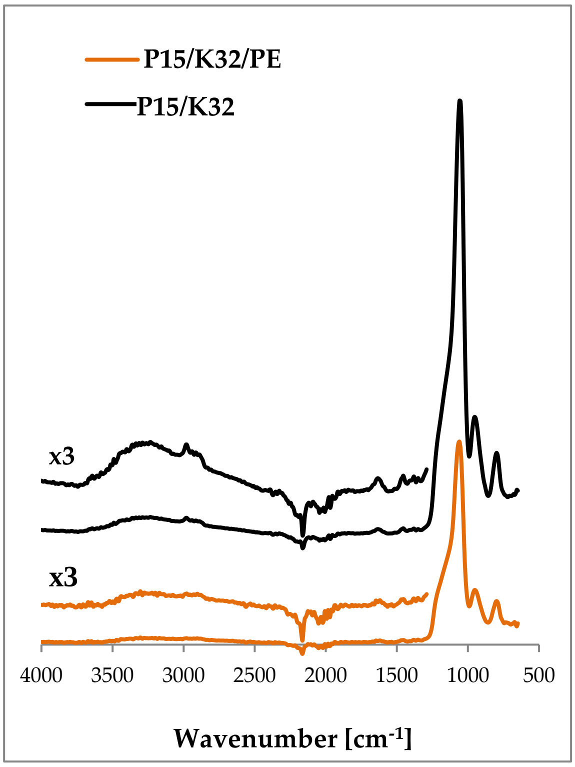

Figure S6. FT-IR spectra of as-synthesized and extracted SBA-15 materials functionalized with Z-6032 aminoorganosilane introduced by co-condensation.

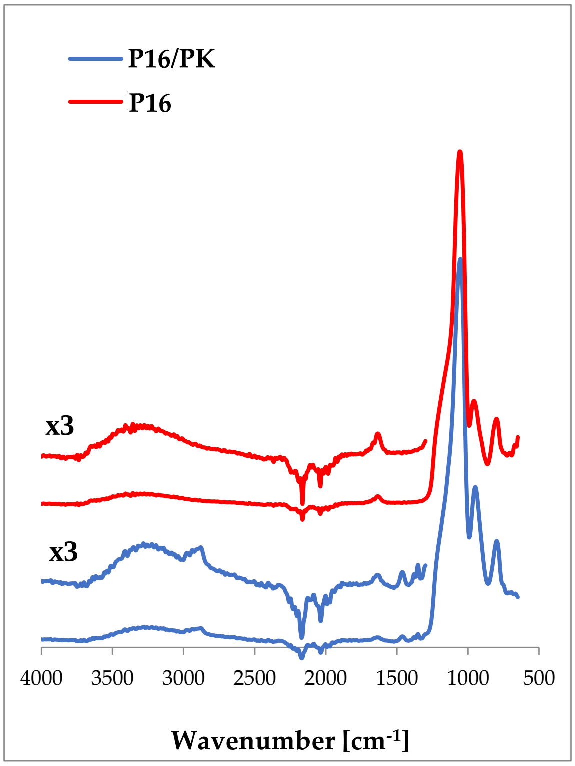

Figure S7. FT-IR spectra of as-synthesized and calcined SBA-16 materials.

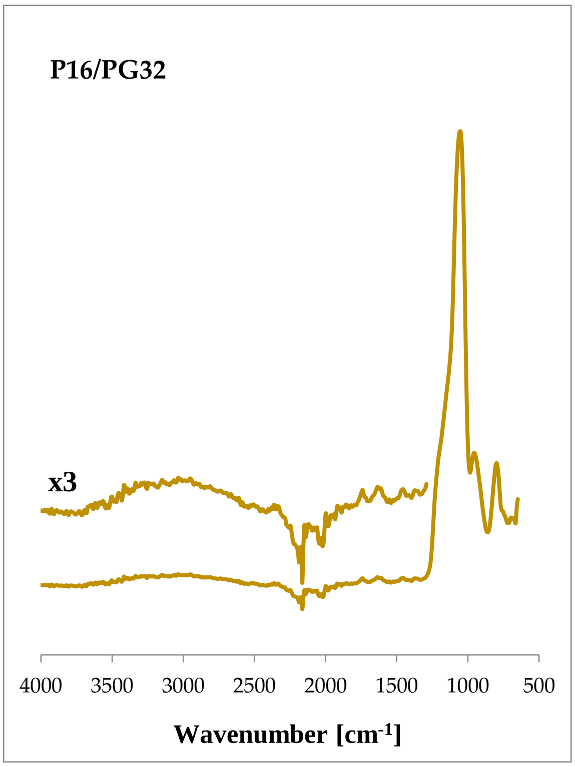

Figure S8. FT-IR spectra of as-synthesized and extracted SBA-16 materials functionalized with Z-6032 aminoorganosilane introduced by co-condensation.

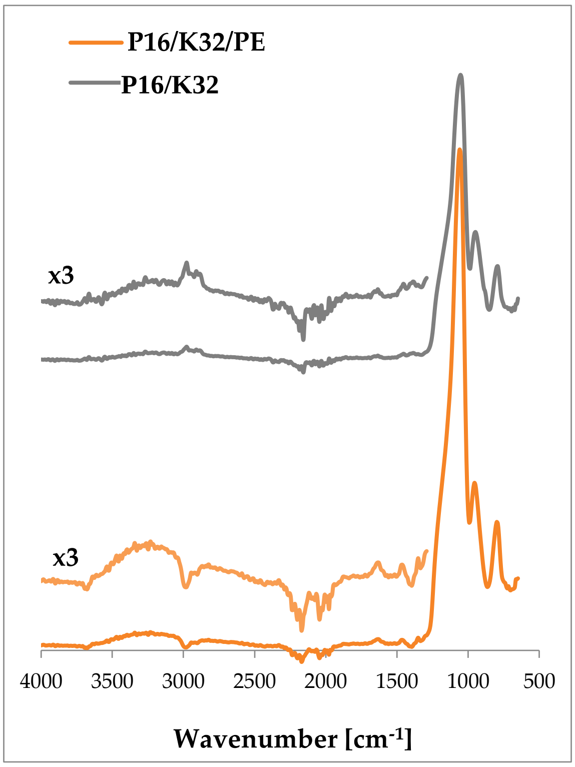

Figure S9. FT-IR spectra of SBA-16 material functionalized by grafting of Z-6032 aminoorganosilane.

A)

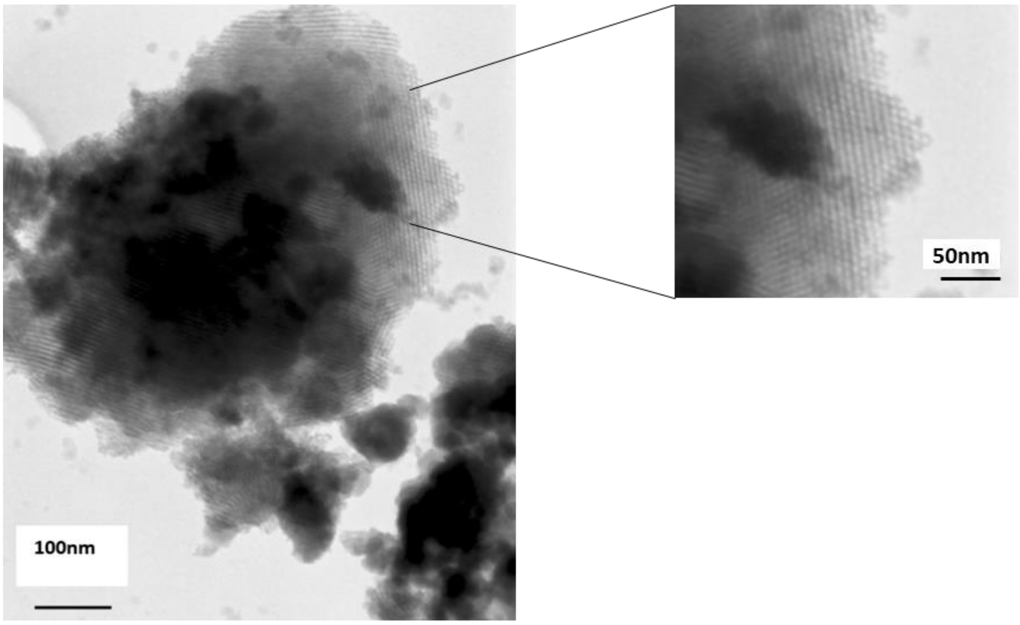

B)

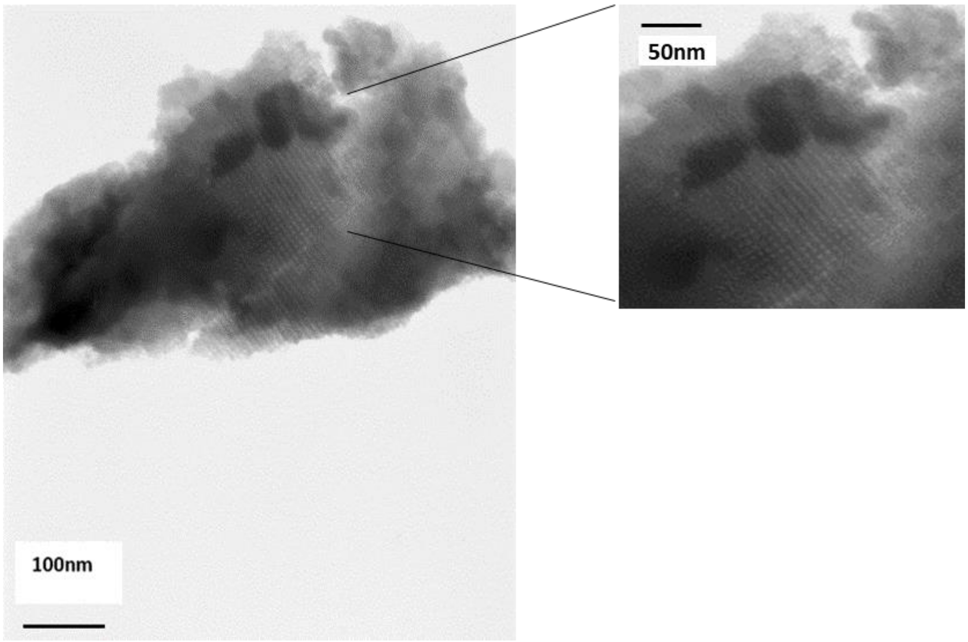

C)

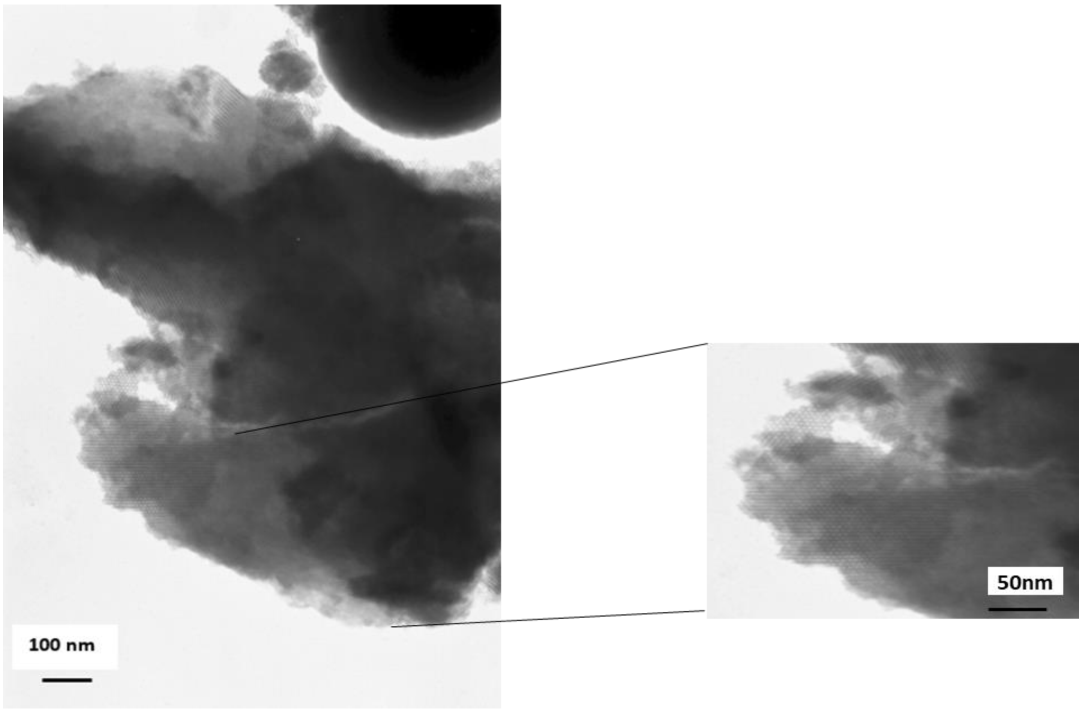

D)

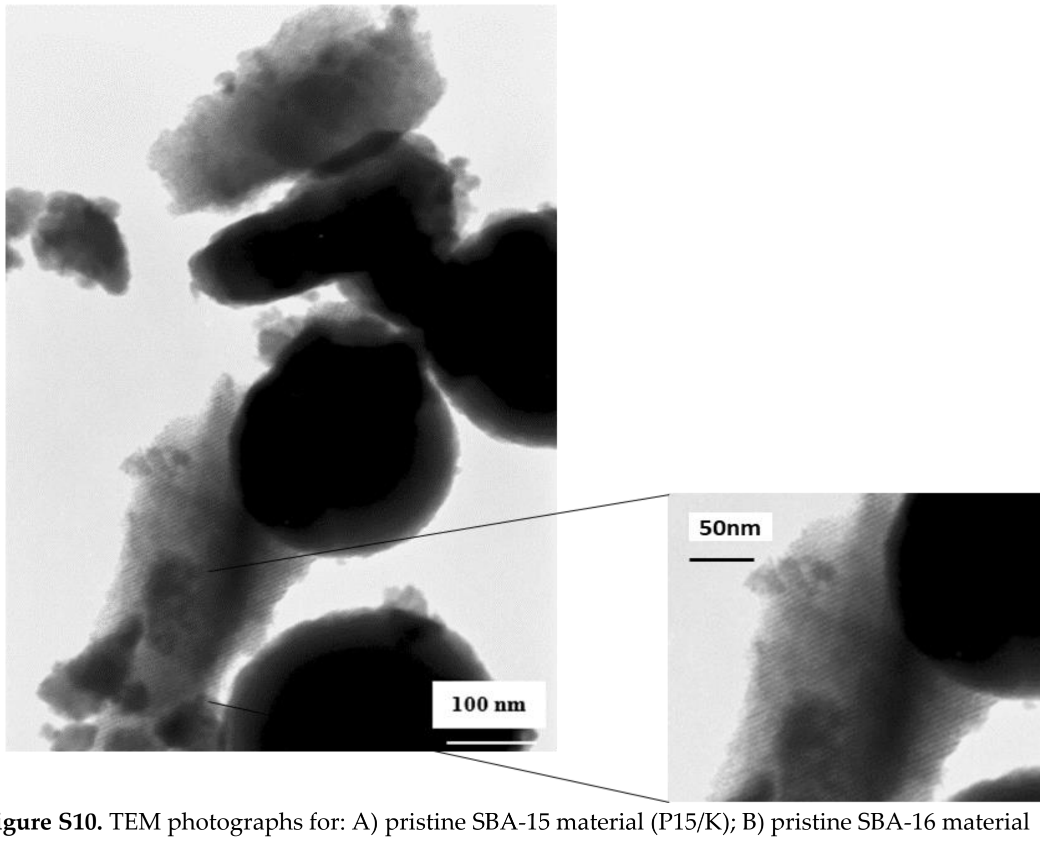

Figure S10. TEM photographs for: A) pristine SBA-15 material (P15/K); B) pristine SBA-16 material (P16/K); C) SBA-15 grafted with Z-6032 (P15/PG32); D) SBA-16 grafted with Z-6032 (P16/PG32).

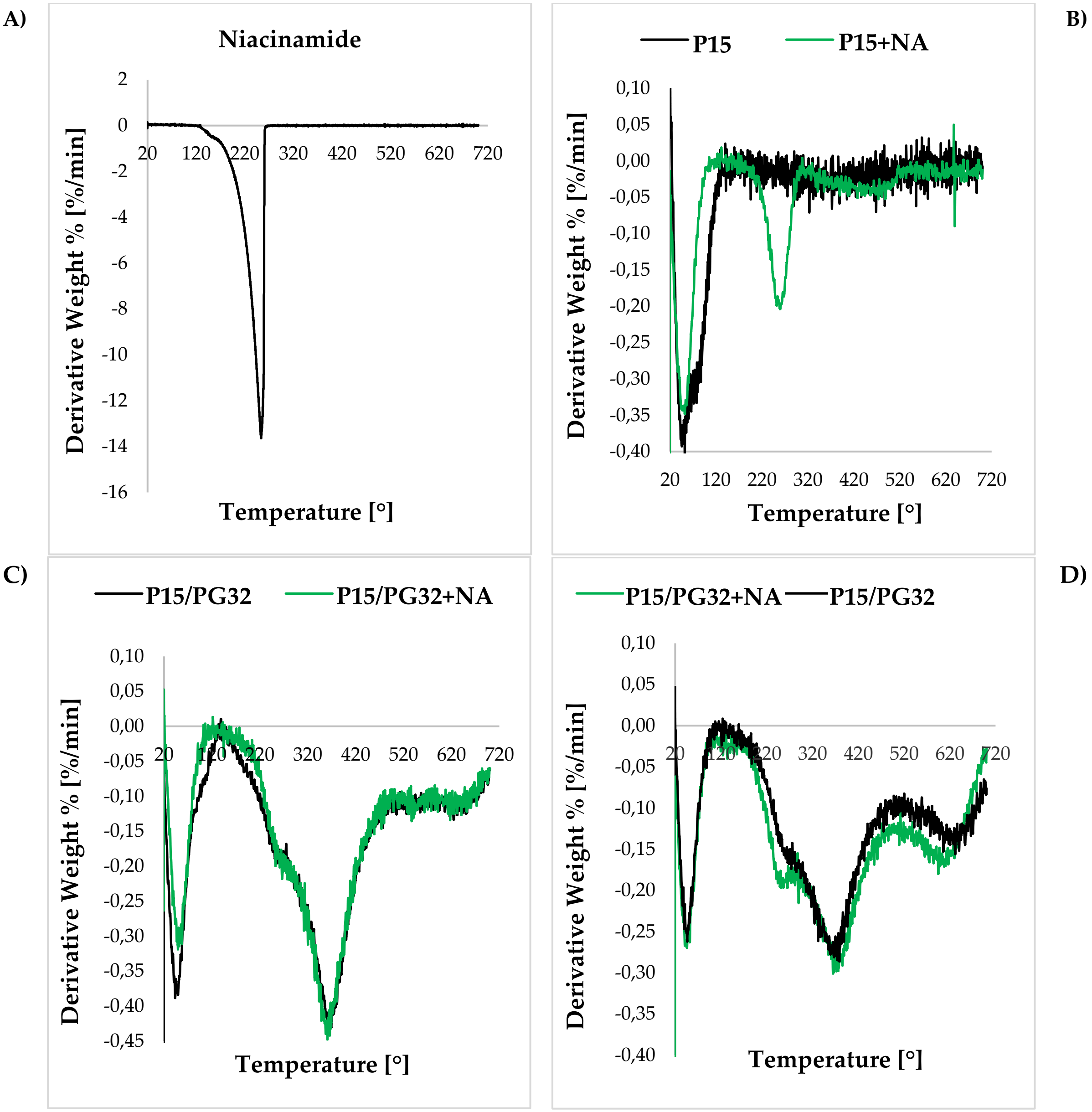

Figure S11. Results of thermogravimetric analysis of: A) niacinamide; B) pristine SBA-15 and loaded with niacinamide; C) SBA-15 modified with aminoorganosilane Z-6032 introduced by co-condensation and loaded with niacinamide; D) SBA-15 modified with aminoorganosilane Z-6032 introduced by grafting and loaded with niacinamide.

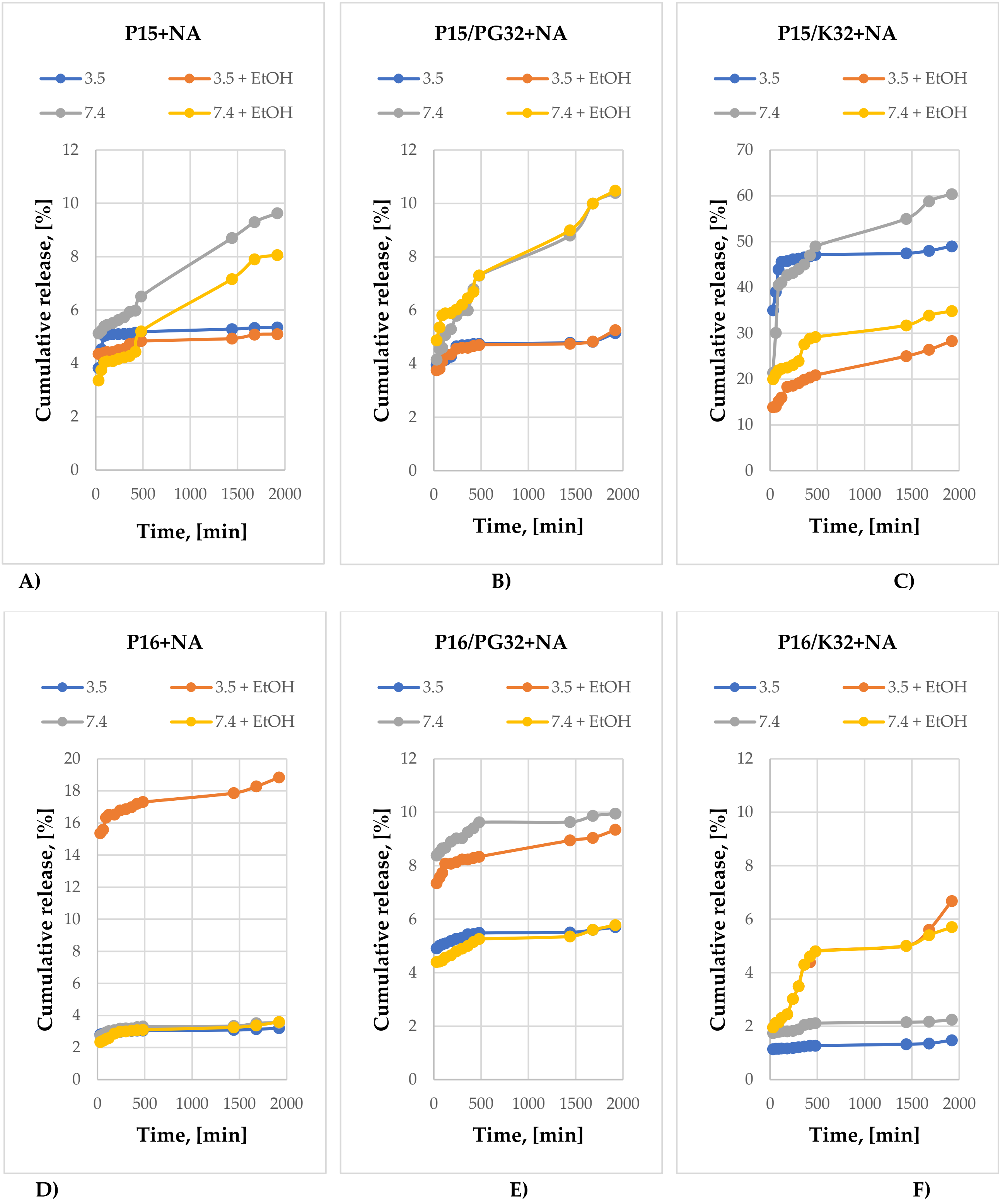

Figure S12. Niacynamide release profiles from mesoporous silica-based materials into acceptor fluids with different pH values.
